# Response to letter to the editor

**DOI:** 10.1590/S1516-31802010000100011

**Published:** 2010-01-07

**Authors:** Hélio Afonso Ghizoni Teive, Salo Haratz, Jorge Zavala, Renato Puppi Munhoz, Rosana Hermínia Scola, Lineu César Werneck

**Affiliations:** 1 MD, PhD. Associate professor, Neurology Service, Department of Internal Medicine, Hospital das Clínicas, Universidade Federal do Paraná (UFPR), Curitiba, Paraná, Brazil.; 2 MD. Resident physician, Neurology Service, Department of Internal Medicine, Hospital das Clínicas, Universidade Federal do Paraná (UFPR), Curitiba, Paraná, Brazil.; 3 MD. Neurologist, Neurology Service, Department of Internal Medicine, Hospital das Clínicas, Universidade Federal do Paraná (UFPR), Curitiba, Paraná, Brazil.; 4 MD, PhD. Head of Neurology Service, Department of Internal Medicine, Hospital das Clínicas, Universidade Federal do Paraná (UFPR), Curitiba, Paraná, Brazil.

Dear Editor,

We thank Professor Wiwanitkit for his interest in our case report entitled Lhermitte’s sign and vitamin B12 deficiency: case report.^1^ Professor Wiwanitkit raised concerns about the final diagnosis of the case presented in our paper.

Professor Wiwanitkit stated that before there are any neurological manifestations in cases of vitamin B12 deficiency, anemia development may be seen and such anemia may present several signs and symptoms. We agree with this comment and, concordantly with this observation, the first symptom reported by our patient was the electric shock-like sensation through his neck and spreading down his spine and lower limbs, triggered by neck flexion [Lhermitte’s sign (LS)]. The purpose of our report was to show that, in rare instances, one of these signs or symptoms of anemia may be LS. Secondly, he states that LS can be seen in other disorders, particularly in the elderly, and this was extensively discussed in the text. In our case report, after the use of intramuscular vitamin B12 therapy, complete recovery was progressively achieved over a one-year period. Moreover, after three years of follow-up, there has not been any malignancy or any sign of other diseases. Multiple sclerosis is a well-known cause of LS, but is rare in this age group and, most importantly, was not detected in our patient, since neither imaging nor cerebrospinal fluid (CSF) studies indicated any possibility of this diagnosis.
